# Inhibition of Aberrant Circulating Tfh Cell Proportions by Corticosteroids in Patients with Systemic Lupus Erythematosus

**DOI:** 10.1371/journal.pone.0051982

**Published:** 2012-12-17

**Authors:** Xuebing Feng, Dandan Wang, Jingjing Chen, Lin Lu, Bingzhu Hua, Xia Li, Betty P. Tsao, Lingyun Sun

**Affiliations:** 1 Department of Rheumatology, The Affiliated Drum Tower Hospital of Nanjing University Medical School, Nanjing, Jiangsu, China; 2 Division of Rheumatology, David Geffen School of Medicine at UCLA, Los Angeles, California, United States of America; University Medical Center Freiburg, Germany

## Abstract

**Objective:**

To observe the proportion of peripheral T follicular helper (Tfh) cells in patients with systemic lupus erythematosus (SLE) and to assess the role of steroids on Tfh cells from SLE patients.

**Methods:**

Peripheral blood mononuclear cells (PBMCs) from 42 SLE patients and 22 matched healthy subjects were collected to assess proportions of circulating CXCR5^+^PD1^+^/CD4^+^ T cells (Tfh), CD4^+^CCR6^+^ T cells (Th17-like) and CD19^+^CD138^+^ plasma cells by flow cytometry. 8 of the patients had their blood redrawn within one week after receiving methylprednisolone pulse treatment. Disease activity was evaluated by SLE disease activity index. To test the effect of IL-21 and corticosteroids on Tfh cells in vitro, PBMCs harvested from another 15 SLE patients were cultured with medium, IL-21, or IL-21+ dexamethasone for 24 hours and 72 hours. PBMCs from an independent 23 SLE patients were cultured with different concentrations of dexamethasone for 24 hours.

**Results:**

Compared to normal controls, percentages of circulating Tfh cells, but not Th17 cells, were elevated in SLE patients and correlated with disease activity. Proportions of Tfh cells in SLE patients were positively correlated with those of plasma cells and serum levels of antinuclear antibodies. After methylprednisolone pulse treatment, both percentages and absolute numbers of circulating Tfh cells were significantly decreased. In vitro cultures showed an increase of Tfh cell proportion after IL-21 stimulation that was totally abolished by the addition of dexamethasone. Both 0.5 and 1 µM dexamethasone decreased Tfh cells dose dependently (overall p = 0.013).

**Conclusions:**

We demonstrated that elevated circulating Tfh cell proportions in SLE patients correlated with their disease activities, and circulating levels of plasma cells and ANA. Corticosteroids treatment down-regulated aberrant circulating Tfh cell proportions both *in vivo* and *in vitro*, making Tfh cells a new treatment target for SLE patients.

## Introduction

Systemic lupus erythematosus (SLE) is a prototype autoimmune disease characterized by the production of multiple autoantibodies, particularly antinuclear antibodies (ANA). Both the B-cell and T-cell compartments exhibit functional abnormalities that could lead to the autoantibody production in SLE. Recently, much attention has focused on the role of the T follicular helper (Tfh) cell in supporting antibody production. This CD4^+^ T subset helps the generation of germinal center (GC) responses where somatic hyper-mutation and affinity maturation take place leading to the generation of memory B cells and plasma cells [1].

Tfh cells can migrate to the germinal center through chemokine receptor CXCR5, while programmed death 1 (PD-1) signaling limits the number of Tfh cells and enhances their ability to promote interleukin (IL)-21 expressions [2]. It has been demonstrated that co-expressions of CXCR5 with ICOS and/or PD-1 are a useful phenotypic profile to distinguish this T helper cell subset, and circulating Tfh cells are in proportion to their GC counterparts [3]–[5]. Growing evidence has suggested that Tfh cell may have a crucial role in the pathogenesis of SLE, as the aberrant expressions of Tfh cell-associated molecules, such as ICOS, SAP, PD-1 and IL-21, are all linked to autoantibody production and/or lupus-like disease in murine models [6]–[9]. In SLE patients, expression of Tfh cell phenotypes by circulating peripheral blood cells has been shown to correlate with diversity and titers of autoantibodies and with disease severity [5].

IL-21, which is mainly expressed by Tfh cells, is crucial for Tfh cell development [10]. Previously we have shown there was a nearly perfect correlation between the extent of IL-21 and ANA production in vitro [11], strongly supporting a role of Tfh cell in the developing of SLE. However, it is still unclear how these cells are modulated in SLE. In this study, we explored the frequency of Tfh cells in peripheral blood from SLE patients, and examined the role of IL-21 and steroids on these cells. We found a high frequency of circulating Tfh cells in lupus patients, especially in those with active disease, which could be alleviated by corticosteroids both *in vivo* and *in vitro*.

## Materials and Methods

### Study Subjects

Study protocol and informed consent were reviewed and approved by the Ethics Committee of the Affiliated Drum Tower Hospital of Nanjing University Medical School and all participants provided their written informed consents to participate in this study. All the SLE patients fulfilled the 1997 updated revised criteria of the American College of Rheumatology (ACR) [12] and healthy volunteers were recruited as normal controls with an effort to match age and gender of SLE patients.

Forty-two SLE patients (35 women and 7 men, mean age 33.7±2.0 years) were enrolled for the measuring of circulating Tfh cells. Disease activity at the time of blood drawn was assessed by their rheumatologists and verified by two of the authors (DW and BH) using the SLE disease activity index (SLEDAI) [13]. Eight of the patients (7 women and 1 man, mean age 27.4±4.1 years) had their blood redrawn after high-dose steroid therapy. The demographics for these patients were shown in Table 1 and Table 2. Of the 42 SLE patients recruited, 29 was reassessed for ANA values, 24 for IgG antibodies to anti-dsDNA and 37 for anti-extractable nuclear antigen using reagents from Euroimmun AG (Lübeck, Germany). Values for ANA were calculated as described [11] and detection range for IgG anti-dsDNA antibody is 10∼800 IU/ml.

**Table 1:** Demographics of SLE patients and normal controls*.

	SLE patients (n = 42)	Normal controls (n = 22)
Age, years	33.7±2.0 (14–62)	34.1±2.2 (21–55)
Female gender	35 (83.3)	20 (90.9)
Disease duration, years	7.05±1.02 (0.25–33)	
SLEDAI score	8.26±0.55 (2–16)	
ANA positivity	100%	

* Values are shown by mean ± SEM (range) or number (percentage). There were no significant differences between patients with systemic lupus erythematosus (SLE) and normal controls or disease controls in terms of age and gender.

**Table 2:** Alteration of Tfh cells before and after MP treatment.

No	Age	Gender	Before MP treatment		After MP treatment	
			Tfh (%)	Tfh num(10^6^/L)	Tfh (%)#	Tfh num(10^6^/L)*
1	29	f	11.26%	56.3	5.48%	43.8
2	25	f	15.31%	275.6	8.25%	173.3
3	37	f	7.33%	161.3	9.51%	190.2
4	22	f	18.5%	166.5	8.62%	43.1
5	49	f	22.46%	202.1	14.16%	141.6
6	14	m	12.24%	195.8	7.67%	115.1
7	29	f	22.43%	314.0	15.29%	183.5
8	14	f	32.5%	617.5	28.83%	403.6

MP: methylprednisolone. Data were analyzed by paired t-test.

# p<0.01 vs Tfh cell percentage before MP treatment,

* p<0.05 vs absolute Tfh cell numbers before MP treatment.

As for in vivo studies, peripheral blood mononuclear cells (PBMCs) were obtained from 15 SLE patients (14 women and 1 man, mean age 34.7±3.1 years) and another 23 samples (21 women and 2 men, mean age 32.9±3.3 years) were collected to observe the dose effect of steroids.

### Flow Cytometry

**Figure 1:**
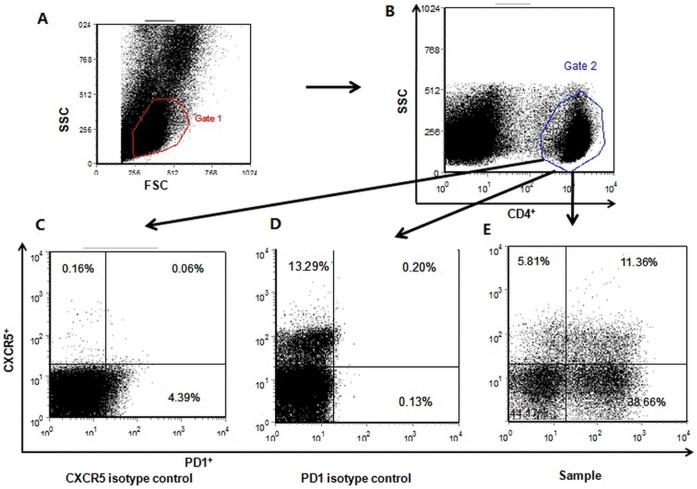
FACS analysis of Tfh cells. Three tubes from one sample, stained with 1) CD4, CXCR5 isotype control and PD1, 2) CD4, CXCR5 and PD1 isotype control, and 3) CD4, CXCR5 and PD1 separately, were analyzed at the same time. PBMCs are gated on the forward scatter of living cells and then centered on CD4^+^ T cells (A,B). After setting the threshold using isotype control staining for CXCR5 (C) and PD1 (D), the proportion of Tfh cells is analyzed as the percentage of CXCR5^+^ PD1^+^ cells within total CD4^+^ T cells (E).

PBMCs were isolated by Ficoll-Paque Plus density gradient centrifugation (Burlington, Onlario, Canada). As described elsewhere [14], PBMCs at 10^6^/tube were stained with fluorescein isothiocyanate (FITC)-conjugated anti-CD4, phycoerythrin (PE)-conjugated anti-PD1, phycoerythrin-Cy5 (PE-Cy5)-conjugated anti-CCR6 and Alexa Fluor 647-conjugated anti-CXCR5 (BD PharMingen San Diego, CA) or isotype-matched control IgG, according to the manufacturer’s protocol. Flow data were determined in a FACSCalibur system (BD Biosciences, San Jose, CA), followed by analysis using Flowjo software (Tree Star, version 5.7.2). At least 50,000 events per sample were analyzed. Cells were gated on the forward scatter of living cells and then centered on CD4^+^ T cells. As illustrated in Figure 1, the proportion of Tfh cells was analyzed as the percentage of CXCR5^+^ PD1^+^ cells within total CD4^+^ T cells for each sample (CXCR5^+^ PD1^+^/CD4^+^ T cells, using isotype control staining for CXCR5 and PD1 to set the threshold). To determine the absolute number of circulating Tfh cells, lymphocyte numbers per liter were obtained by automated complete blood cell counts at the same day blood drawn and then multiplied by percentages of CD4^+^ and percentages of CXCR5^+^ PD1^+^/CD4^+^ cells. CD4^+^ CCR6^+^ T cells proportion was defined as Th17-like cells as previously described [15]. For the detection of circulating plasma cells, PBMC were stained with APC-conjugated anti-CD19 and PE-conjugated anti-CD138, and gating on CD19^+^ CD138^+^ cells [16]. Data were reported as CD19^+^ CD138^+^ percentages of total PBMCs.

### Cell Culture

PBMCs were collected by Ficoll-Paque Plus density gradient, resuspended in RPMI 1640 medium, then distributed to 96-well plates (Corning, Tewksbury, MA) at (0.5 −1.5)×10^6^ per well. Cells incubated at 37°C in a humidified atmosphere with 5% CO_2_ were treated as follows: (a) in RPMI-1640 medium containing 10% FCS alone, (b) recombinant human IL-21 (200 ng/µL) (PeproTech, Rocky Hill, NJ) added, or (c) IL-21 and dexamethasone (1 µM, as described before [17]) (Jinlin pharmacy, China) added simultaneously. Experiments were carried out in duplicates and cell numbers were equal among the 3 groups for each patient. After 24 and 72 hours, all the cells in culture wells were harvested and then stained with FITC-conjugated anti-CD4, PE-conjugated anti-PD1 and Alexa Fluor 647-conjugated anti-CXCR5 for the detection of Tfh cell percentage by flow cytometry. To observe the role of dexamethasone on unstimulated Tfh cells, PBMCs were cultured in RPMI-1640 medium containing 10% FCS with or without different concentrations of dexamethasone (0.5 µM, 1 µM) for 24 hours. To analyze the status of cell apoptosis after dexamethasone treatment, cultured cells were harvested, stained with PE-conjugated anti-CD4, PE-Cy7-conjugated anti-PD1, APC-conjugated anti-CXCR5 and FITC-conjugated Annexin V, and then measured by FACS.

### Statistical Analysis

To compare numeration data between two groups from *in vivo* studies, Mann-Whitney U test was conducted because some of the data were not normally distributed and values were shown as medians with 25th and 75th percentiles and interquartile range (IQRs), except that alterations of Tfh cells before and after methylprednisolone treatment were analyzed by paired t-test. Chi-square test or Fisher exact probability test was applied for quantitative data, and Pearson correlation was used to depict linear relationships between two factors. For our *in vitro* studies, paired t-test was applied to compare results between two groups and Kruskal-Wallis test was used to determine the difference among three groups. Data were analyzed using the Prism 3.0 program (GraphPad, La Jolla, CA, USA) and SPSS 16.0 software, and p<0.05 was considered significant.

## Results

### Percentages of Circulating CXCR5^+^ PD1^+^/CD4^+^ T cells Increased in SLE Patients and Correlated with Disease Activity

**Figure 2:**
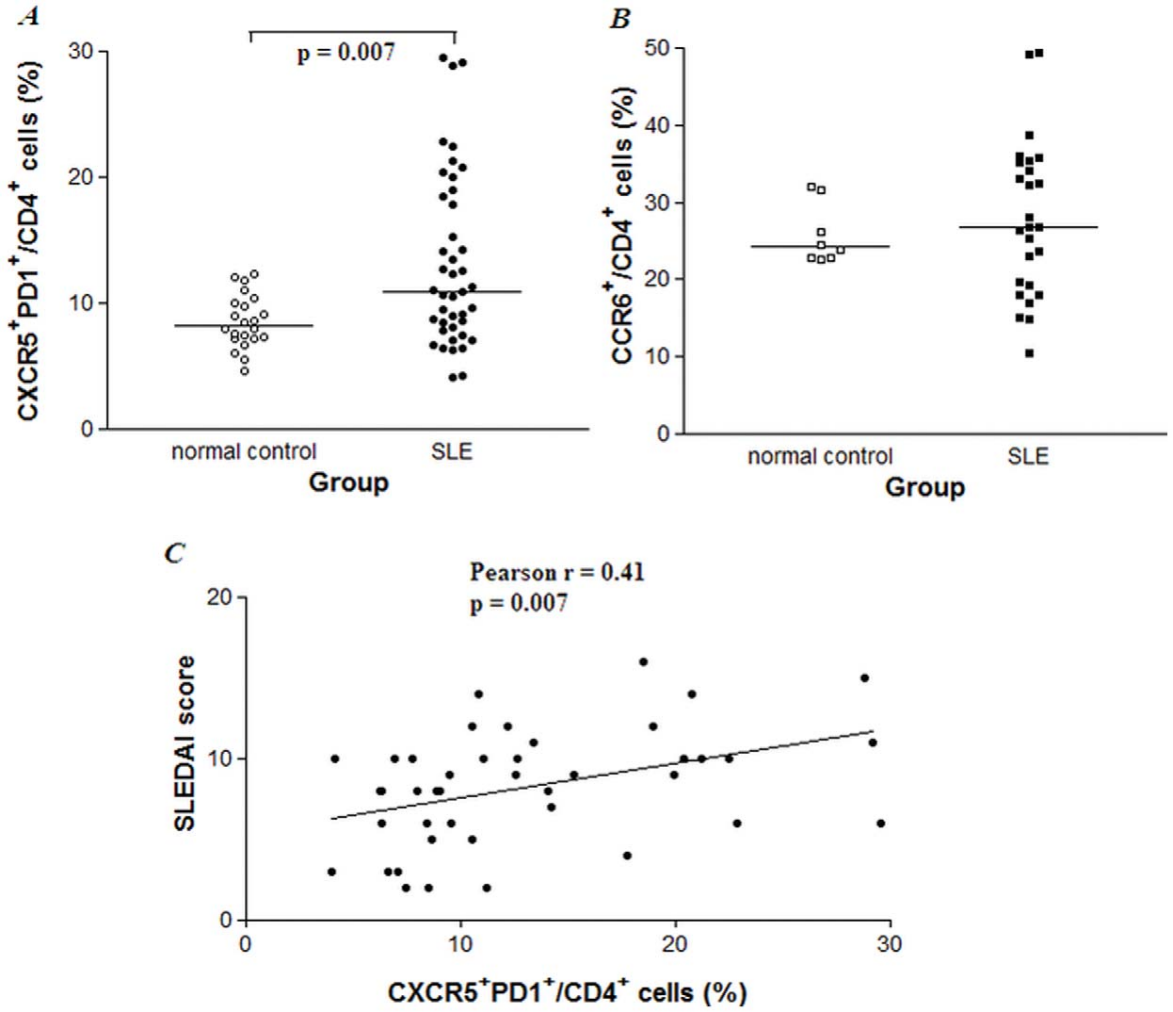
Aberrant circulating CXCR5^+^ PD1^+^/CD4^+^ but not CCR6^+^/CD4^+^ T cells correlated with disease activity in SLE patients. A. Elevated circulating CXCR5^+^ PD1^+^/CD4^+^ cells in SLE patients compared to normal controls. PBMCs were quantified by FACS, each symbol represented an individual sample and horizontal lines showed median values. Mann-Whitney U test was conducted to compare the data between two groups. B. Circulating CCR6^+^/CD4^+^ cell proportions enriched for Th17-like cells were compared between SLE patients and normal controls. C. Correlation between percentages of CXCR5^+^ PD1^+^/CD4^+^ cells and disease activity of 42 SLE patients. Pearson correlation was applied to depict linear relationships.

Percentages of peripheral blood CD4^+^ CXCR5^+^ PD1^+^ cells in CD4^+^ T cells from 42 Chinese SLE patients and 22 normal controls were analyzed by flow cytometry. Compared with the normal controls, the SLE patients were not significantly different in terms of age and gender (Table 1). As shown in Figure 2A, percentages of CXCR5^+^ PD1^+^/CD4^+^ cells were higher in peripheral blood samples from SLE patients compared with those from normal controls (median 10.94 (25th and 75th percentiles 8.11, 18.32) % vs. 8.17 (7.20, 9.93) %, p<0.01). To assess whether T helper type 17 (Th17) cells, another subset of CD4^+^ T cells involved in IL-21 and subsequent antibody production, played a role in SLE, peripheral blood CD4^+^ CCR6^+^ cells from 26 SLE patients and 8 normal controls were measured concordantly. As shown in Figure 2B, the proportion of circulating CD4^+^ CCR6^+^ in CD4^+^ T cells showed no difference between SLE patients (26.75 (19.33, 34.83) %) and healthy controls (24.15 (22.78, 27.53) %, p>0.05). Next we compared percentages of circulating CXCR5^+^ PD1^+^/CD4^+^ cells in SLE patients with varying levels of disease activity, as assessed by the SLEDAI score at the time blood was obtained. A positive correlation between Tfh cells and SLEDAI scores was observed (Pearson r = 0.41, p<0.01) (Figure 2C).

### Correlation of Tfh cell Proportions with Levels of Autoantibodies and Plasma Cell Proportions in SLE Patients

**Figure 3:**
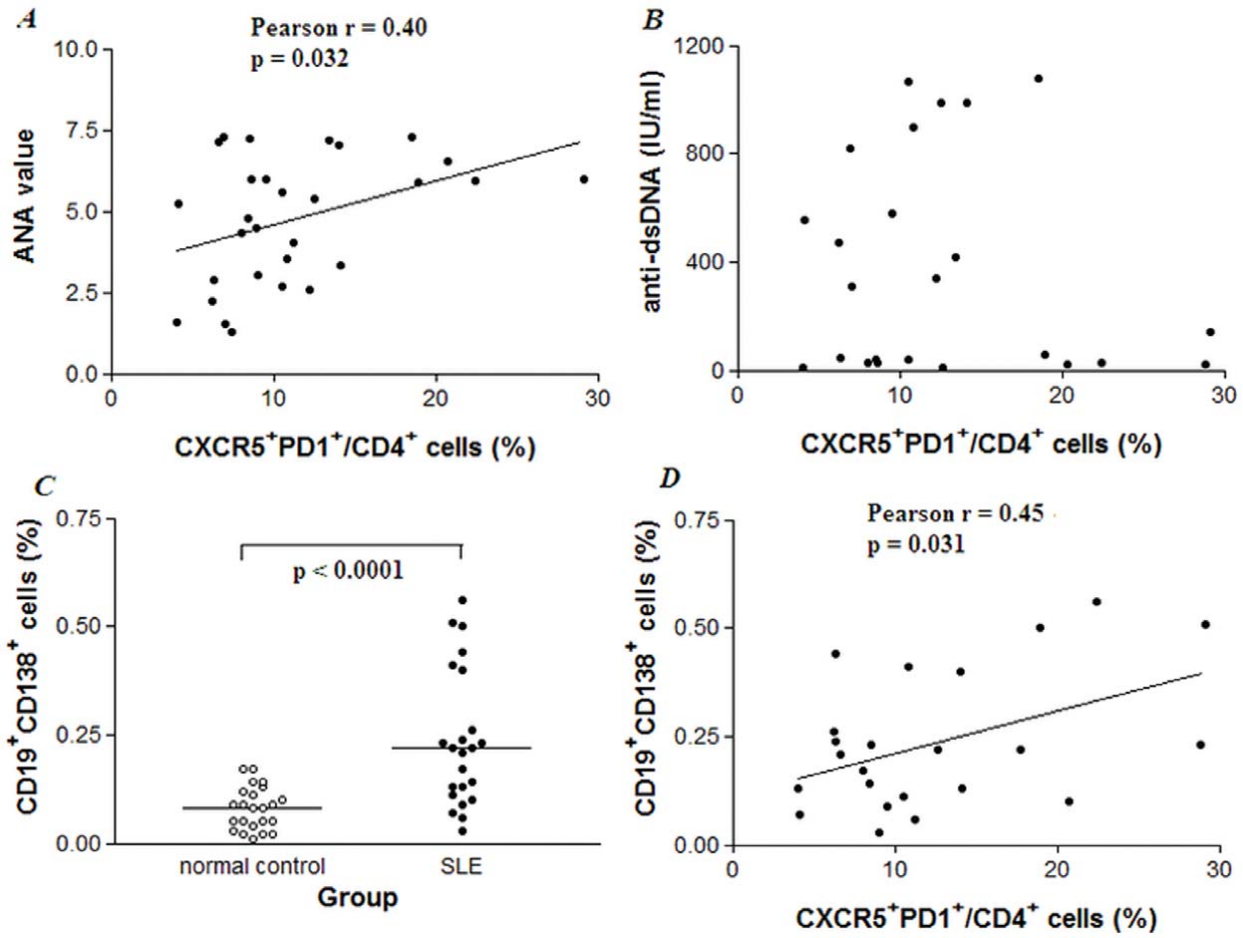
Positive correlation of CXCR5^+^ PD1^+^/CD4^+^ cells with ANA and CD19^+^ CD138^+^ cells in SLE patients. A. Percentages of circulating CXCR5^+^ PD1^+^/CD4^+^ cells were positively correlated with ANA values (Pearson r = 0.40, p<0.05) in 29 SLE patients. B. There were no association between percentages of CXCR5^+^ PD1^+^/CD4^+^ cells and circulating levels of IgG anti-dsDNA by Pearson correlation test. However, in those patients having high levels of anti-dsDNA antibodies (∼ 50%), a trend of correlation with CXCR5^+^ PD1^+^/CD4^+^ cells was observed. C. Circulating CD19^+^ CD138^+^ cells were elevated in SLE patients as compared with normal controls. Each symbol represented an individual sample, horizontal lines showed median values, and Mann-Whitney U test was applied. D. Percentages of CXCR5^+^ PD1^+^/CD4^+^ cells were positively correlated with percentages of CD19^+^ CD138^+^ cells in 23 SLE patients (Pearson r = 0.45, p<0.05).

Within our 42 patients, all had a positive documented ANA. 29 of their samples were measured for ANA levels and 24 for anti-dsDNA levels by ELISA using serum collected at the time of blood draw. Percentages of CXCR5^+^ PD1^+^/CD4^+^ cells were correlated with ANA levels in these patients (Pearson r = 0.40, p = 0.032) (Figure 3A), but not correlated with levels of IgG anti-dsDNA antibodies (Figure 3B). In 37 patients who were routinely checked for anti-extractable nuclear antigen (ENA) antibodies, only anti-SSA/SSB was associated with Tfh cell proportion as shown in Table 3 (p<0.05).

**Table 3:** Correlation between clinical manifestations and Tfh cells in SLE patients*.

Clinical manifestations	Circulating Tfh cells		p
	High expression#	Low expression	
Mucocutaneous	5/16 (31.3)	9/26 (34.6)	0.82
Renal involvement$	12/16 (75.0)	17/26 (65.4)	0.73
Renal insufficiency	4/16 (25.0)	5/26 (19.2)	0.71
CNS lupus	1/16 (6.3)	2/26 (7.7)	1
Serositis	5/16 (31.3)	3/26 (11.5)	0.22
Vasculitis	4/16 (25.0)	1/26 (3.8)	0.061
Leucopenia	4/16 (25.0)	5/26 (19.2)	0.71
Thrombocytopenia	5/16 (31.3)	3/26 (11.5)	0.22
Hypocomplementemia	8/16 (50.0)	15/26 (57.7)	0.63
Autoantibodies			
Anti-RNP	3/13 (23.1)	10/24 (41.7)	0.31
Anti-SSA/SSB	9/13 (69.2)	8/24 (33.3)	0.047
Anti-Sm	3/13 (23.1)	7/24 (29.2)	1

* Values were the number of patients positive/total number tested (%) and Chi-square test or Fisher exact probability test was applied to compare results between two groups.

# Defined as having a percentage of CXCR5^+^ PD1^+^/CD4^+^ cells 2 SD above the mean of normal controls.

$ Defined as having proteinuria, hematuria or urinary casts.

The link between Tfh cell and ANA prompted us to test the status of B cell activation in SLE patients. Flow cytometry analysis revealed a significant higher proportion of circulating CD19^+^ CD138^+^ plasma cells in 23 SLE patients (0.22 (0.12, 0.33) %) compared to those in 23 healthy controls (0.08 (0.05, 0.12) %, p<0.0001) (Figure 3C), and percentages of CD19^+^ CD138^+^ cells was positively correlated with percentages of CXCR5^+^ PD1^+^/CD4^+^ cells in SLE patients (Pearson r = 0.45, p = 0.031) (Figure 3D).

### Tfh cell and SLE Clinical Features

Renal involvement, serositis, leucopenia, thrombocytopenia, hypocomplementemia, and ongoing rash as well as oral ulcer contributed to SLEDAI scores in most of our SLE patients, and 3 of them had central nervous system (CNS) involvement. Patients were divided into high Tfh group (n = 16, defined as having a percentage of CXCR5^+^ PD1^+^/CD4^+^ cells 2 standard deviations above the mean of normal controls) and normal Tfh group (n = 26). Based on our data (Table 3), all the manifestations listed above were not associated with percentages of Tfh cells. Interestingly, there was a trend showing patients with high Tfh expression had elevated incidences of vasculitis (25% vs 3.8%, p = 0.06).

### Decreased Proportion of Tfh cells after Methylprednisolone Pulse Treatment in SLE Patients

All recruited patients had been treated with different dose of corticosteroids (<60 mg of methylprednisolone per day) before blood drawn. Subsequently, 8 of them were given high dose of intravenous methylprednisolone (120–500 mg/day for 3–4 days) to control their active disease (including 2 CNS lupus, 5 lupus nephritis and 1 severe thrombocytopenia), which provided us an excellent opportunity to explore the role of corticosteroids in Tfh cell *in vivo*. To evaluate the alteration of circulating CXCR5^+^ PD1^+^/CD4^+^ cells, blood was redrawn from these 8 patients within one week after the pulse treatment. As shown in Table 2, concomitantly with the amelioration of clinical manifestations, there was a significant reduction of circulating CXCR5^+^ PD1^+^/CD4^+^ cell proportion post-treatment (p<0.01). To exclude the influence of corticosteroids on total lymphocyte numbers, absolute numbers of Tfh cells pre- and post- treatments were enumerated. Our data showed that absolute number of CXCR5^+^ PD1^+^/CD4^+^ cells was also decreased after the treatment (p<0.05, Table 2).

### Inhibition of IL-21 Stimulated Tfh cell of by Dexamethasone in vitro

**Figure 4:**
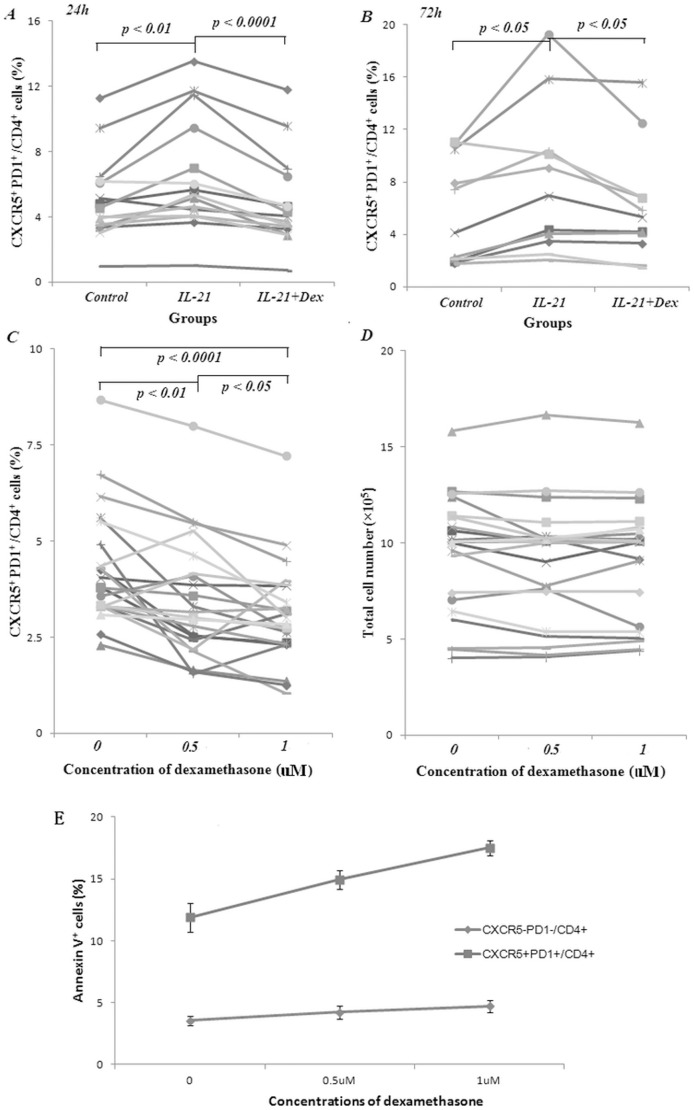
Decreased CXCR5^+^ PD1^+^/CD4^+^ cell production by dexamethasone. A. Dexamethasone (Dex) reduced the proportion of IL-21 stimulated CXCR5^+^ PD1^+^/CD4^+^ cell. Each line represented the alterations of CXCR5^+^ PD1^+^/CD4^+^ cell percentages of an individual sample when cultured with medium control, IL-21 or IL-21+ Dex for 24 hours in vitro. Paired t-test was applied to compare results between two groups and Kruskal-Wallis test was used to determine the difference among three groups. Compared with control group, CXCR5^+^ PD1^+^/CD4^+^ cells were increased when cultured with IL-21 (p<0.01). Adding dexamethasone to IL-21 culture system significantly inhibited the production of CXCR5^+^ PD1^+^/CD4^+^ cells (p<0.0001). B. A similar tendency was observed when cells were cultured for 72 hours. Each line represented the alterations of CXCR5^+^ PD1^+^/CD4^+^ cell percentages of an individual sample. C. Dexamethasone reduced the proportion of CXCR5^+^ PD1^+^/CD4^+^ cell in a dose-dependent manner. Each line represented the changes of CXCR5^+^ PD1^+^/CD4^+^ cell percentages of an individual sample when cultured with different concentrations of dexamethasone for 24 hours in vitro and paired t-test was applied to compare results between two groups. D. Downregulation of CXCR5^+^ PD1^+^/CD4^+^ cell by dexamethasone was not a consequence of the decline in total cell numbers. Cells were equally divided into 3 groups to culture with different concentrations of dexamethasone. After 24 hours culturing, all the cells were harvested and counted by flow cytometry. There were no differences of total cell numbers among the groups (p>0.05). E. Increased cell apoptosis after dexamethasone treatment. PBMCs from 6 SLE patients were collected and cultured in the presence or absence of dexamethasone for 24 hours. Then cells were harvested and quantified by FACS. Data were shown as mean ± SEM. The percentages of Annexin V^+^ cells are much higher in CXCR5^+^ PD1^+^/CD4^+^ groups, especially for those treated with high dose dexamethasone.

To further elucidate the role of IL-21 and steroids in Tfh cell production, PBMCs from another 15 randomly selected SLE patients were collected. Each sample was divided into 3 groups: medium control group, IL-21 stimulation group and IL-21+ dexamethasone group, cultured for 24 hours and harvested to measure CXCR5^+^ PD1^+^/CD4^+^ cells by flow cytometry. We found percentages of CXCR5^+^ PD1^+^/CD4^+^ cells significantly increased after IL-21 stimulation (p<0.01) which could be totally abolished by adding dexamethasone (p<0.0001) (Figure 4A). Similar results were observed after culturing these PBMCs for 72 hours (p<0.05) (Figure 4B).

### Dose Dependent Effects of Dexamethasone on Tfh cell

PBMCs were collected from 23 SLE patients and each sample was divided into 3 parts to cultured with different concentrations of dexamethasone (0, 0.5 µM and 1 µM, defined as control, low dose and high dose) for 24 hours and then measured for CXCR5^+^ PD1^+^/CD4^+^ cells. We found the percentages of CXCR5^+^ PD1^+^/CD4^+^ cells significantly decreased after culturing with dexamethasone (p<0.01 for low dose group and p<0.0001 for high dose group as compared to control group, and overall p value among 3 groups were 0.013 by Kruskal-Wallis test). Percentages of CXCR5^+^ PD1^+^/CD4^+^ cells were lower in the high dose group comparing to those in the low dose group (p<0.05), indicating a dose dependent effect of dexamethasone on Tfh cell (Figure 4C). To address whether the down-regulation of Tfh cells by dexamethasone was due to a concentration-related decrease in cell numbers, total cells in each group were counted and there was no difference among the groups (p>0.05, Figure 4D). Next we evaluated the status of cell apoptosis after dexamethasone treatment by using Annexin V staining. Our data showed that Annexin V^+^ cells were increased both in CXCR5^+^ PD1^+^/CD4^+^ group and in CXCR5^−^PD1^−^/CD4^+^ group when treated with dexamethasone, especially for those using high dosage. However, the percentages of Annexin V^+^ cells were much high in CXCR5^+^ PD1^+^/CD4^+^ groups (Figure 4E).

## Discussion

To our knowledge, this is the first paper to report the effect of corticosteroids in the aberrant expression of Tfh cells in SLE patients. Our study showed that proportions of the CXCR5^+^ PD1^+^/CD4^+^ T (Tfh) cells in peripheral blood of SLE patients were elevated compared to those in normal subjects, exhibiting positively correlations with disease activity score, peripheral plasma cells as well as ANA titers (Figure 2 and 3). Tfh cell numbers dramatically reduced after the patients received methylprednisolone pulse treatment (Table 2). Adding IL-21 to PBMC cultures could increase Tfh cell numbers *in vitro*, which was down-regulated when cocultured with dexamethasone in a dose-dependently manner (Figure 4), suggesting that Tfh cells could be a new therapeutic target for autoimmune disease.

Consistent with ours [11], several studies have also provided evidences that the IL-21 pathway plays an important role in the pathogenesis of SLE [18], [19]. The production of IL-21 is mainly restricted to two subsets of CD4^+^ T cells, Th17 and Tfh. Th17 cells are associated with the production of inflammation mediators, and it has been observed by several groups that SLE patients have an enhanced Th17 cell response [20], [21], which is different from our results showing that there is no elevation of CD4^+^ CCR6^+^ cell percentage in PBMCs from SLE patients as compared to that from normal controls. Because cell types other than Th17, such as Th22, could also express CCR6, we measured CD3^+^ CD8^−^ IL-17A^+^ cells in PBMCs after stimulation by FACS and found no difference in their proportions between SLE patients and normal controls (data not shown). Since more than 90% of IL-17-secreting CD4^+^ T cells were also CCR6^+^ cells [22], it was reasonable to assume that CD4^+^ CCR6^+^ could represent Th17-like cells. Although the sample size in this study was not large enough to draw a conclusion, evidences have shown that increased Th17 cells in SLE patients were linked to the use of corticosteroids [23], implicating an abnormal Th17 response in SLE might be derived from treatments rather than the disease itself. Consistent with our findings, Dolff et al has shown percentages of IL-21^+^ T cells were increased in SLE patients, while percentages of IL-17^+^ T cells were not [18]. Recently, it has been shown that both CXCR5^+^ CD4^+^ T cells and CXCR5^−^ CD4^+^ T cells (Th17 related) producing IL-21 were increased in SLE patients. However, only the former cell subset was correlated with increased circulating germinal center B cells [24].

In addition to Th17 cells, another subset of CD4^+^ T cells named Tfh cells could also produce massive IL-21 [25]. IL-21 has been considered as the master cytokine for the regulation of Tfh development and function in an autocrine fashion [26]. Here we found proportions of circulating Tfh cells were elevated in SLE patients and correlated with disease activity as measured by SLEDAI scores, suggesting Tfh cells but not Th17 cells might be the main producer of IL-21 in SLE that contribute to the disease processes. To support our results, frequencies of cells displaying Tfh phenotypes have been shown to increase in peripheral blood and spleens of human SLE patients and lupus-prone mice [5], [27]. No association of Tfh cell expression with renal, CNS or hematological involvement was observed in our study. However, an increased tendency of vasculitis was suggested for those with high Tfh cell expression (Table 3).

The involvement of Tfh cells in pathogenic mechanisms of SLE remains to be demonstrated. Tfh cells express higher levels of co-stimulatory molecules like OX40, CD40L, ICOS and PD-1 as compared to other T cell subsets, thus could provide co-stimulation signal to activate antigen presenting B cells and consequently exuberance in the positive selection of somatically mutated B cells will lead to excessive autoantibody production [28], [29]. In addition, Tfh cells are capable of secreting cytokines (such as IL-21 and IL-6) to drive B cell expansion and differentiation [3]. In this study, we showed significantly positive correlation of proportions of Tfh cells with long-lived plasma cells in SLE patients. Similar to that reported [5], proportion of Tfh cells was also associated with ANA and anti-SSA antibody production, supporting the notion that Tfh cells may interact with B cells to facilitate plasma cells differentiation and antibody production.

Our next question is whether Tfh cells could be modulated by current treatments. Corticosteroids are still the mainstay of treatment for SLE patients, which can decrease pro-inflammatory cytokine synthesis, T-cell function, and Fc receptor expression, thereby controlling inflammation and resolving lupus activity [30], [31]. For those patients with severe organ involvement (for example, cardiopulmonary, renal or CNS involvement), usually high-dose of corticosteroids are given to control disease activity. Previously it has been shown the expansion of Tfh cells in peripheral blood is a fixed phenotype and not related to immunosuppressive therapy [5]. Here we observed a significant reduction in the Tfh cells after methylprednisolone pulse treatment. Considering there was a slight decline of total lymphocyte numbers after corticosteroid treatment (data not shown), absolute Tfh cell numbers were calculated and a similar result was shown. To confirm this finding, *in vitro* studies were performed and we found Tfh proportions were significantly decreased when dexamethasone added to the culture system.

The underlying mechanisms for corticosteroid in modulating Tfh cells and its relation to IL-21 are still unclear. As reported, Tfh cells could be differentiated or transformed from other T cell types under suitable conditions, and IL-21 was a key cytokine to induce Tfh production [10]. In this paper, we have shown that IL-21 stimulation did increase Tfh cell proportion, but the effect diminished when dexamethasone was added to the culture system. To interpret this phenomenon, cell apoptosis after *in vitro* culturing was measured by using Annexin V staining. Our data showed that Annexin V^+^ cells were increased both in CXCR5^+^ PD1^+^/CD4^+^ group and in CXCR5^−^ PD1^−^/CD4^+^ group when treated with dexamethasone, but apoptotic cells were much higher in CXCR5^+^ PD1^+^/CD4^+^ groups (Figure 4E). Based on these findings, it is plausible that IL-21 helps induce Th cell differentiation to Tfh subtypes, while corticosteroid promotes Tfh cell apoptosis. Combined effects of these two factors may counteract each other, resulting in unchanged Tfh cell percentages. In addition, corticosteroid may directly regulate Tfh related cytokines including IL-21 and IL-6 via serum glucocorticoid-regulated kinase 1 (SGK1), an important gene in steroids-related pathway [32], [33].

Taken together, here we showed the dynamics of circulating Tfh cells in Chinese SLE populations for the first time. Our results indicated that Tfh cells might interact with B cells and facilitated autoantibodies production in SLE. The proportion of circulating Tfh cells was down-regulated by corticosteroids in a dose-dependent manner, thus providing us a new target in the treatment of SLE patients.
